# COVID-19 Vaccine Uptake in Undocumented Latinx Patients Presenting to the Emergency Department

**DOI:** 10.1001/jamanetworkopen.2024.8578

**Published:** 2024-04-26

**Authors:** Jesus R. Torres, Breena R. Taira, Angela Bi, Andrew V. Gomez, Cynthia Delgado, Stephanie Vera, Robert M. Rodriguez

**Affiliations:** 1 David Geffen School of Medicine, University of California, Los Angeles; 2 Olive View-UCLA Medical Center, Los Angeles, California; 3 School of Medicine, University of California, San Francisco

## Abstract

This cross-sectional study examines COVID-19 infection status, vaccination uptake, and perceptions about the vaccine among Latinx patients presenting to the emergency department in California.

## Introduction

COVID-19 has disproportionately affected the US Latinx population, with significantly higher rates of infections, hospitalizations, and mortality.^1,2^ Although the undocumented Latinx population in the US is growing,^3^ there are limited data regarding their COVID-19 infection status, vaccination uptake, and perceptions about the COVID-19 vaccine.

## Methods

We conducted this prospective, cross-sectional survey study from September 2, 2021, through March 31, 2022, at Olive View-UCLA Medical Center and San Francisco General Hospital, which serve large Latinx immigrant populations. After verbal consent, as approved by the institutional review boards of both facilities, a convenience sample of adult (aged ≥18 years) emergency department (ED) patients participated in structured interviews in English or Spanish. The study followed the STROBE reporting guideline.

We excluded patients who were critically ill, on psychiatric holds, or incarcerated or who had altered mentation. Participants were enrolled in 3 approximately equal groups based on self-reported race and ethnicity (non-Latinx [including Asian, Black, Native Hawaiian or other Pacific Islander, Middle Eastern, White, and other], legal Latinx resident or citizen, and undocumented Latinx).

Descriptive statistics were used to summarize participant responses, and comparisons were performed using bivariate hypothesis testing. Data were analyzed using Stata, version 16.1 (StataCorp LLC). Additional details on methodology and the survey instrument are provided in the eMethods in Supplement 1.

## Results

This study included 306 participants (median [IQR] age, 51 [40-60] years; 146 female [48%], 157 male [51%], and 3 with missing sex data [1%]); 209 identified as Latinx (68%) and 34 as non-Latinx Black (11%), 43 as non-Latinx White (14%), and 20 as other race or ethnicity (7%). Among the undocumented Latinx group, 26 (25%) were uninsured, 99 (95%) reported Spanish as their primary language, and 31 (30%) used the ED as their usual source of health care (Table 1).

**Table 1.** Patient Characteristics

Characteristic	No. of patients (%)			
	All (N = 306)	Undocumented Latinx (n = 104)	Documented	
			Legal Latinx resident (n = 105)	Non-Latinx (n = 97)^a^
Age, median (IQR), y	51 (40-60)	47 (38-57)	50 (39-60)	57 (45-65)
Sex				
Female	146 (48)	52 (50)	59 (56)	35 (36)
Male	157 (51)	52 (50)	44 (42)	61 (63)
Missing	3 (1)	NA	NA	NA
Have primary care	201 (66)	60 (58)	73 (70)	68 (70)
Health insurance type				
Private	17 (6)	1 (1)	7 (7)	9 (9)
Medicare	23 (8)	1 (1)	10 (10)	12 (12)
Medicaid	138 (45)	44 (42)	50 (48)	44 (45)
Kaiser Permanente	3 (1)	NA	NA	3 (3)
My Health Los Angeles or Healthy San Francisco	48 (16)	21 (20)	15 (14)	12 (12)
Other	16 (5)	NA	6 (6)	10 (10)
Uninsured	40 (13)	26 (25)	10 (10)	4 (4)
Missing	21 (7)	NA	NA	NA
Primary language				
English	112 (37)	3 (3)	31 (30)	78 (80)
Spanish	173 (57)	99 (95)	73 (70)	1 (1)
Other	21 (7)	2 (2)	1 (1)	18 (19)
Homeless	19 (6)	6 (6)	5 (5)	8 (8)
Emergency department is usual source of care	78 (25)	31 (30)	25 (24)	22 (23)
Received an influenza vaccine in last 5 y	180 (59)	60 (58)	71 (68)	49 (51)

Abbreviation: NA, not available.

^a^ Includes participants identifying as Asian (11 [4%]), Black (34 [11%]), Native Hawaiian or other Pacific Islander (2 [1%]), Middle Eastern (4 [1%]), White (43 [14%]), or other (3 [1%]).

Compared with the non-Latinx group, a prior COVID-19 infection was more likely reported by undocumented Latinx participants (odds ratio [OR], 3.42; 95% CI, 1.66-7.23) and legal Latinx residents (OR, 2.73; 95% CI, 1.32-5.83). Of all participants, 265 (87%) reported having received at least 1 COVID-19 vaccine and 41 (13%) reporting a decline in vaccination, with similar distributions for all 3 groups. The most common reason for declining vaccines was concern about potential side effects (15 participants [37%]). Compared with the undocumented Latinx group, the non-Latinx group was less likely to believe that undocumented immigrants could receive the COVID-19 vaccine in the US (OR, 0.09; 95% CI, 0.01-0.39), and 41 (13%) reported knowing undocumented people who did not obtain the vaccine because of fear of discovery and deportation (Table 2). Among those who previously declined a COVID-19 vaccine, 9 (22%) expressed interest in receiving the vaccine while in the ED.

**Table 2.** COVID-19 Vaccine Uptake and Perceptions

Survey item	No. of patients (%)			
	All	Undocumented Latinx	Legal Latinx resident	Non-Latinx
COVID-19 infection and vaccine, No.	306	104	105	97
Previously had a COVID-19 infection	90 (29)	40 (38)	35 (33)	15 (16)
Received ≥1 COVID-19 vaccine	265 (87)	91 (88)	89 (85)	85 (88)
Declined vaccine	41 (13)	13 (13)	16 (15)	12 (12)
Reasons for COVID-19 vaccine hesitancy, No.	41	13	16	12
Worried about side effects	15 (37)	3 (23)	6 (38)	6 (50)
Did not believe that COVID-19 vaccines work	7 (17)	3 (23)	2 (13)	2 (17)
Did not have access to the internet	6 (15)	2 (15)	2 (13)	2 (17)
Did not have an identification card	5 (12)	NA	2 (13)	3 (25)
Other reasons	8 (20)	5 (39)	4 (25)	4 (33)
Believed that undocumented immigrants can get the COVID-19 vaccine in the US, No.	276	74	105	97
Yes	242 (88)	72 (97)	96 (91)	74 (76)
No	8 (3)	2 (3)	1 (1)	5 (5)
Unsure	26 (9)	NA	8 (8)	18 (19)
Know undocumented immigrants who are afraid of getting the COVID-19 vaccine because they think they might be reported to the authorities, No.	305	104	105	96
Yes	41 (13)	17 (16)	16 (15)	8 (8)
No	264 (87)	87 (84)	89 (85)	88 (92)

Abbreviation: NA, not applicable.

## Discussion

Among the population studied, a prior COVID-19 infection was most commonly reported by Latinx participants. Despite the health care access barriers undocumented Latinx individuals face,^4^ we found no differences in the proportion who had received COVID-19 vaccines. Additionally, there were minimal perceptions of exclusion from eligibility for vaccination among this group. However, 13% of all participants knew people who were reluctant to get vaccinated due to concerns about their immigration status.

Limitations of our work include group differences in age, sex, and insurance status, factors that may have influenced their responses to key questions. Additionally, citizenship status and prior COVID-19 infection were self-reported, and we only sampled patients who sought care in the ED; thus, individuals who were most fearful of discovery of their immigration status may have been undercounted. Our study was also restricted to 2 EDs affiliated with teaching hospitals in California; therefore, generalizability may be limited. Overall, our findings highlight the utility of EDs for greater inclusion of the undocumented immigrant population in public health surveillance research.

## Notes

Supplement 1.
eMethods.

eReferences

Supplement 2.
Data Sharing Statement
