# Supplementary material for: COVID-19 Vaccine Uptake in Undocumented Latinx Patients Presenting to the Emergency Department
Source: JAMA Netw Open. 2024 Apr 26;7(4):e248578. doi: 10.1001/jamanetworkopen.2024.8578 (PMC11053375; doi:10.1001/jamanetworkopen.2024.8578)
Supplement: Supplement 1. — eMethods. eReferences [file jamanetwopen-e248578-s001.pdf]

Supplemental Online Content

Torres JR, Taira BR, Bi A, et al. COVID-19 vaccine uptake in undocumented Latinx patients presenting to the emergency department. *JAMA Netw Open*. 2024;7(4):e248578. doi:10.1001/jamanetworkopen.2024.8578

eMethods.

eReference

This supplemental material has been provided by the authors to give readers additional information about their work.

© 2024 Torres JR et al. *JAMA Network Open.*

eMethods

Survey Instrument Development

Subject matter experts from the University of California Los Angeles and the University of California San Francisco created an initial survey, both in English and Spanish. Study investigators pilot tested the initial survey with 5 Emergency Department (ED) patients who provided feedback on the wording, length, and clarity, to ensure its cultural and linguistic appropriateness and accuracy. The 16-point survey was finalized based on the feedback received.

Research Assistants

Research assistants (RA), fluent in both English and Spanish, underwent over 4 hours of observed survey implementation training by the study investigators. RAs screened the ED electronic health record for patients during a 2-4 hour block, between 0700 to 2200, 7 days a week, depending on their availability.

Selection of Participants

We enrolled a convenience sample of English- and Spanish-speaking adult ED patients (≥ 18 years of age) at varying hours of the day on different days of the week. We sought to enroll approximately 150 participants (50 in the Undocumented Latinx group, 50 in the Legal Latinx resident group, 50 in the Non-Latinx) at each site, or approximately 300 total, based on RA availability during our study period and based on our prior research.[1] Once one group contained 20 fewer participants than the other two groups, RAs utilized screening information to enroll more participants in that particular group. During the RA availability block, all patients in the

© 2024 Torres JR et al. *JAMA Network Open.*

ED during that time frame were screened via the electronic health record ED board. Those that met inclusion criteria were approached after they had received an initial assessment by their treatment team (medical screening exam) in the ED, to ensure clinical stability. After scripted verbal consent, RAs administered the survey verbally in either English or Spanish, in a participant's private clinical treatment area to ensure confidentiality.

Key questions and measures

The key measure of interest was having received a COVID-19 vaccine previously. COVID-19 vaccine received was defined as a "Yes" and vaccine not received as a "No" to the following question, "Have you received a COVID-19 vaccine yet?" Secondary key measures focused on having had a previous COVID-19 infection and legal status. Having had a previous COVID-19 infection was defined as a "Yes" to the following question, "Have you previously been diagnosed with COVID-19 or had a positive COVID-19 test?". In our demographics section of the survey, we assessed a measure for undocumented status by asking, "Are you a legal resident/citizen of the United States?", which has been previously employed.[1]

Data Analysis

We used descriptive statistics to summarize participant responses. We presented data using odds ratios and 95% confidence intervals and utilized 2-sided bivariate hypothesis testing at α = 0.05 significance level.

© 2024 Torres JR et al. *JAMA Network Open.*

eReference

1. Maldonado CZ, Rodriguez RM, Torres JR, Flores YS, Lovato LM. Fear of discovery among latino immigrants presenting to the emergency department. Academic Emergency Medicine. 2013 Feb;20(2):155–61.

© 2024 Torres JR et al. *JAMA Network Open.*

ED Based COVID-19 Prevention Program Survey____

Date of Survey_________

1. Have you previously been diagnosed with COVID-19 or had a positive COVID-19 test?
   a. Yes
   b. No
   c. Unsure

   1A. **(If YES to 1)** which month/year_____________

2. Have you heard about the COVID-19 vaccines (vaccines to prevent COVID-19)?
   a. Yes
   b. No
   c. Unsure

3. Have you received a COVID-19 vaccine yet?
   a. Yes
   b. No

3A. **(If YES to 3)** when did you get it? (What month/year)_____________

3B. **(If YES to 3)** where did you get it?
   a. Mass vaccination clinic
   b. Here at the hospital
   c. An ER
   d. From my doctor
   e. At a pharmacy
   f. Other ____

3C. **(IF YES to 3)** Do you know which vaccine you received?
   a. Pfizer
   b. Moderna
   c. Johnson and Johnson
   d. I have received one but I do not know which

3D. **(IF YES to Pfizer or Moderna in 3C.)** Did you get both doses?
   a. Yes
   b. No,
      i. if no, why not?____________________
   c. I plan on getting my second dose soon

APPROVED
September 2, 2021
OV-UCLA ERI
Institutional Review Board

---

4. (**If NO on question 3)** Have you wanted to get the COVID vaccine?

A. Yes
B. No
C. Unsure

4A. (**If YES to 4)** If you have wanted to get the COVID-19 vaccine, what is/are the reasons that you have not gotten it?

a. I didn't know where to get it
b. I was afraid that I would have to give too much information when I get the vaccine
c. I didn't know that I was eligible
d. I don't have an identification card (ID) or Driver's License
e. I don't have a primary care doctor
f. I don't have internet to sign up for one
g. I don't have a car or transportation to get to a place where I can get it
h. I have been too busy
i. I thought it would be too expensive (if respondent says this, tell them that they are free)
j. I don't speak English
k. OTHER___________________________

4B. **(If answered NO or UNSURE to 4)** why haven't you wanted to get a COVID-19 vaccine?

a. I have already had COVID-19 (if they answer this, tell them that they should still get it)
b. I am worried about side effects and safety of the vaccine
c. I don't believe that the COVID-19 vaccine will work
d. I am not worried about getting COVID-19
e. I need more information about the vaccine
f. I have heard stories on media (online, social media, television, or radio) that give me doubts about vaccines
g. I thought it would be too expensive (if respondent says this, tell them that they are free)
h. People that I know tell me I shouldn't get it
i. OTHER ___________________________

5A. **If NO to question 3** *AT SITES WHERE YOU* ***CAN*** *GET A VACCINE IN THE ED THAT DAY*:

COVID-19 vaccines are available free here in the ED. While these vaccines cannot assure complete protection, they will decrease your and your family members' likelihood of becoming infected with COVID-19. Over 300 million doses have been given to people in the United States and are extremely safe.

Will you accept the vaccine today during your ED visit?

A. Yes

APPROVED
September 2, 2021
OV-UCLA ERI
Institutional Review Board

B. No
C. Unsure

If yes they want the vaccine, tell the provider that the patient wants a COVID vaccine.

5B. **If NO to question 3** *AT SITES WHERE YOU **CANNOT** GET A VACCINE IN THE ED THAT DAY*:

COVID-19 vaccines are available free to you at a number of sites in Los Angeles. While these vaccines cannot assure complete protection, they will decrease your and your family members' likelihood of becoming infected with COVID-19. Over 300 million doses have been given to people in the United States and are extremely safe.

Provide them with information about where they can get the vaccine

Will you go to get the vaccine at one of these places?

A. Yes
B. No
C. Unsure

---

6. Do you live with anyone?

a. Yes
b. No

6A. (**If YES to 6**) have any of the people you live with received the COVID-19 vaccine?

a. Yes, all of them
c. Yes, some of them
d. No, none of them
e. Unsure

7. Have you had a flu vaccine in the past 5 years?

a. Yes
b. No
c. Unsure

7A. (**If YES to 7**) where did you get this flu vaccine? (pick all that apply)

a. My doctor's office or clinic
b. A pharmacy
c. An ER - emergency department
d. An urgent care clinic (not my doctor or clinic)
e. Other_________________
f. I don't remember

8. What is your age: ____

ED Based COVID-19 Prevention Program Survey

Date of Survey_________

8A. What is your gender? (check one)

a. Male
b. Female
c. Trans Male
d. Trans Female
e. Genderqueer/Gender Non- binary
f. Decline to answer

9. What is your race/ethnicity? (check all that apply)

a. African-American/Black

b. Asian

c. Hispanic/Latinx

d. Middle Eastern/Chaldean

e. Native American /American Indian

f. Native Hawaiian or Pacific Islander

g. White (non-Latinx)

h. Other_________

i. Decline to answer

10. Do you currently have a place to live:

a. Yes
b. No

10A. **(If No to 10)** how long have you been homeless?

a. < 1 month
b. 1-6 months
c. 6 months – 1 year
d. > 1 year

11. What is your primary language?

a. English
b. Spanish
c. Cantonese/Mandarin
d. Tagalog
e. Arabic

APPROVED
September 2, 2021
OV-UCLA ERI
Institutional Review Board

f. Armenian
g. Farsi
h. Korean
i. Russian
j. Vietnamese
k. Other: _________________________

11A**.** (**If English is NOT your primary language from 11**) how well do *you* speak and understand English?

a. Not at all
b. A little
c. Most of it
d. All of it (completely)

12. Do you have health insurance: ☐ Yes ☐ No ☐ I am currently applying for it ☐ Unsure

12A. (**If YES to 12**) what type? ☐ Private ☐Medicare ☐ MediCal ☐ Kaiser ☐ Affordable Care Act (ObamaCare) ☐ Military of Veterans Administration ☐ My Health LA ☐ Other

13. Do you have a regular clinic or doctor for medical care? ☐ Yes ☐ No

13A. (**If YES to 13**) when was the last time you saw <u>*this*</u> doctor or went to the clinic?

- < 1 month
- 1-6 months
- 6 months – 1 year
- > 1 year

13B. (**If NO to 13**) when was the last time you saw <u>*any*</u> doctor or had health care in the U.S. (besides today)?

- < 1 month
- 1-6 months
- 6 months – 1 year
- > 1 year
- I have never seen a doctor in the U.S.

13C. (**If NO to 13**) where do you usually go when you are sick or need medical advice?

- An emergency department
- A clinic
- Urgent care center
- Other__________________________

14. Are you a legal resident/citizen of the United States?

APPROVED
September 2, 2021
OV-UCLA ERI
Institutional Review Board

ED Based COVID-19 Prevention Program Survey

Date of Survey_________

a. Yes
b. No
c. Unsure

14A. **(If NO or UNSURE to 14)** How long have you lived in the United States?

- 6 months or less
- 6 months – 1 year
- > 1 year-5 years
- > 5 years – 10 years
- Over 10 years

15. (**If YES to 14**) Do you believe undocumented immigrants can get COVID vaccines in the United States?
a. Yes
b. No
c. Unsure

15A. (**If NO or UNSURE to 14**) Has being an undocumented immigrant made it more difficult for you to get a COVID vaccine?
a. Yes, a lot more difficult
b. Yes, somewhat more difficult
c. Yes, a little more difficult
d. No, not at all difficult

15B. (**If YES to 15A**) How has it made it more difficult?
a. I don't have an Identification Card (ID) or Driver's License
b. I am afraid that they will ask me questions about my immigration status
c. I am afraid that I will have to give them too much information
d. I don't have a primary doctor or clinic
e. I don't have a car or transportation
f. I don't know where to get it
g. I don't have internet access to schedule an appointment
h. I am afraid that my immigration prospects will be affected if I get the vaccine
i. I am afraid I will get reported to the immigration authorities
j. Other:____

16. Do you know any undocumented immigrants who are afraid of getting the COVID vaccine because they think they might be reported to the authorities?
a. Yes
i. If yes, how many?_____________
b. No

APPROVED
September 2, 2021
OV-UCLA ERI
Institutional Review Board

ED Based COVID-19 Prevention Program Survey___

Date of Survey_________

If they have misconceptions, explain to them that undocumented immigrants can freely get COVID vaccines, that they do not ask about immigration status and do not report to immigration authorities.

APPROVED
September 2, 2021
OV-UCLA ERI
Institutional Review Board

ED Based COVID-19 Prevention Program Survey ____

Date of Survey_________

1. ¿Ha sido diagnosticado previamente con COVID-19 o tuvo una prueba de COVID-19 positiva?
    a. Si
    b. No
    c. No se

   1 A. **(If YES to 1)** qué mes / año _____________

2. ¿Ha oído hablar de las vacunas COVID-19 (vacunas para prevenir COVID-19)?
    a. Si
    b. No
    c. No se

3. ¿Ha recibido ya una vacuna COVID-19?
    a. Si
    b. No

   3A. **(If YES to 3)** ¿cuándo lo obtuvo? (Qué mes / año) _________________

   3B. **(If YES to 3)** ¿dónde lo consiguió?
    a. Clínica de vacunación masiva
    b. Aqui en el hospital
    c. Sala de emergencias
    d. De mi doctor(a)
    e. En una farmacia
    f. Otro ____

   3C. (**If YES to 3**) ¿Sabe qué vacuna recibio?
    a. Pfizer
    b. Moderna
    c. Johnson and Johnson
    d. Sí, he recibido uno pero no sé cuál

   3D. **(IF YES to Pfizer or Moderna in 3C)** ¿A recibido las dos dosis?
    a. Si
    b. No,
        i. If no, ¿Por qué no?_____________________________
    c. Planeo recibir mi segunda dosis pronto

APPROVED
September 2, 2021
OV-UCLA ERI
Institutional Review Board

ED Based COVID-19 Prevention Program Survey

Date of Survey_________

4. **(If NO on question 3),** ¿Ha querido recibir una vacuna de COVID?
   a. Si
   b. No
   c. No se

4.A (**If YES to 4)** Si ha querido recibir la vacuna COVID-19, ¿cuáles son las razones por las que no la ha recibido?
   a. No se donde conseguirlo
   b. Temo tener que dar demasiada información cuando me aplique la vacuna
   c. No sabía que era eligible
   d. No tengo una tarjeta de identificación (ID) ni una licencia de conducer
   e. No tengo doctor(a) de atencion primaria/cabecera
   f. No tengo internet para registrarme
   g. No tengo coche ni transporte para llegar a un lugar donde pueda conseguirlo
   h. He estado demasiado ocupado(a)
   i. Pensé que sería demasiado caro (*if respondent says this, tell them that they are free*)
   j. Yo no hablo ingles
   k. otra ____

4B. **(If answered NO or UNSURE to 4)**¿Por qué no quiere una vacuna COVID-19?
   a. Ya tuve COVID-19 (*if they answer this, tell them that they should still get it*)
   b. Me preocupan los efectos secundarios y la seguridad de la vacuna.
   c. No creo que la vacuna COVID-19 funcione
   d. No me preocupa contraer COVID-19
   e. Necesito más información sobre la vacuna.
   f. He escuchado historias (en línea, redes sociales, televisión o radio) que me dan dudas sobre las vacunas.
   g. Pensé que sería demasiado caro (if respondent says this, tell them that they are free)
   h. La gente que conozco me dice que no debería vacunarme
   i. Otra ____

5A. **If NO to question 3** *AT SITES WHERE YOU **CAN** GET A VACCINE IN THE ED THAT DAY*:

Las vacunas de COVID-19 están disponibles gratis aquí en el la sade de emergencias. Las vacunas no pueden garantizar una protección completa, per si disminuirán la probabilidad de que usted y los miembros de su familia se infecten con COVID-19. Se han administrado más de 300 millones de dosis a personas en los Estados Unidos y son extremadamente seguras.

¿Aceptará la vacuna hoy durante esta visita en la sala de emergencia?
   a. Si
   b. No

APPROVED
September 2, 2021
OV-UCLA ERI
Institutional Review Board

c. No se

If yes they want the vaccine, tell the provider that the patient wants a COVID vaccine.

5B. **If NO to question 3** *AT SITES WHERE YOU **CANNOT** GET A VACCINE IN THE ED THAT DAY*:

Las vacunas COVID-19 están disponibles de forma gratuita en varios lugares de Los Ángeles. No se puede garantizar una protección completa, pero si disminuirán la probabilidad de que usted y los miembros de su familia se infecten con COVID-19. Se han administrado a más de 300 millones de dosis en los Estados Unidos y son extremadamente seguras.

Provide them with information about where they can get the vaccine

¿Irás a ponerte la vacuna en uno de estos lugares?

a. Si

b. No

c. No se

---

6. ¿Vive con alguien?

a. No
b. Si

6A. (**If YES to 6)** ¿Alguna de las personas con las que vive ha recibido la vacuna COVID-19?

a. Si, todos
b. Si, algunos
c. No, ninguno
d. No se

7. ¿Se ha vacunado contra la gripe/influenza en los últimos 5 años?

a. Si
b. No
c. No se

7A. (**If YES to 7**) ¿dónde obtuvo la vacuna contra la gripe/influenza? (elija todo lo que corresponda)

a. El consultorio o la clínica de mi médico
b. Una farmacia

APPROVED
September 2, 2021
OV-UCLA ERI
Institutional Review Board

c. Una sala de emergencias - departamento de emergencias
d. Una clínica de atención de urgencia (no mi médico o clínica)
e. Otro_________________
f. No recuerdo

8. ¿Cuál es su edad? _______
   8A. ¿Cuál es tu género? (marque uno)
   a. Hombre
   b. Mujer
   c. Hombre trans
   d. Mujer trans
   e. Genderqueer / género no binario
   f. Se niega a contester

9. ¿Cuál es su raza / etnicidad? (marque todo lo que corresponda)
   a. Afroamericano / Negro
   b. Asiático
   c. Hispano / Latinx
   d. Medio Oriente / Caldeo
   e. Nativo americano / indio americano
   f. Nativo de Hawái o de las islas del Pacífico
   g. Blanco (no latinx)
   h. Otro_________
   i. Negarse a contester

10. ¿Tiene actualmente un lugar para vivir?
   a. Si
   b. No

   10A. **(If No to 10)** ¿cuánto tiempo ha estado sin hogar?
   a. < 1 mes
   b. 1-6 meses
   c. 6 meses – 1 año
   d. > 1 año

11. ¿Cuál es su idioma principal?
a. Inglés
b. Español
c. Cantonés / mandarín
d. Tagalo
e. Arábe
f. Armenio
g. Farsi
h. Coreano

APPROVED
September 2, 2021
OV-UCLA ERI
Institutional Review Board

Date of Survey_________

i. Ruso
j. Vietnamita
k. Otro: ________________________

11A. (**If English it NOT the primary language from 11**) ¿a qué nivel habla y comprende el inglés?

a. Nada
b. Un poco
c. La mayor parte
d. Todo (completamente)

12. ¿Tiene seguro médico?

- Sí
- No
- Lo estoy solicitando actualmente
- No estoy seguro

12.A **(If YES to 12),** ¿qué tipo?

- Privado
- Medicare
- MediCal
- Kaiser
- Affordable Care Act (ObamaCare)
- Military of Veterans Administration
- My Health LA
- Otro

13. ¿Tiene una clínica o un médico habitual para recibir atención médica?

a. Si
b. No

13A (**If YES to 13**)

En caso afirmativo, ¿cuándo fue la última vez que vio a este médico o fue a la clínica?

- < 1 mes
- 1-6 meses
- 6 meses – 1 año
- > 1 año

13 B. (**If NO to 13**) ¿cuándo fue la última vez que vio a un médico o recibió atención médica en los Estados Unidos. (Además de hoy)?

APPROVED
September 2, 2021
OV-UCLA ERI
Institutional Review Board

ED Based COVID-19 Prevention Program Survey

Date of Survey________

- < 1 mes
- 1-6 meses
- 6 meses – 1 año
- > 1 año
- Nunca fui a ver a un medico en los Estados Unidos

13 C. (**If NO to 13**) ¿a dónde va cuando está enfermo o necesita atencion médica?

- ☐ Un departamento de emergencias
- ☐ Una clinica
- ☐ Centro de atención urgente
- ☐ Otro_________________________

14. ¿Es usted un residente legal / ciudadano de los Estados Unidos?
    a. Si
    b. No
    c. No se

14A. **(If NO or UNSURE to 14)** ¿Cuánto tiempo lleva viviendo en los Estados Unidos?

- 6 meses o menos
- 6 meses – 1 año
- > 1 año - 5 años
- > 5 año - 10 años
- Mas de 10 años

15. (**If YES to 14**) ¿Cree que los inmigrantes indocumentados pueden recibir las vacunas COVID en los Estados Unidos?
    a. Si
    b. No
    c. No se

15A. (**If NO or UNSURE to 14**) ¿Ser un inmigrante indocumentado le ha hecho difícil obtener la vacuna COVID?

a. Si, mucho mas dificil
b. Si, algo mas dificil
c. Si, un poco mas dificil
d. No, nada dificil

15B. (**If YES to 15A**) ¿Cómo se lo ha hecho más difícil?

APPROVED
September 2, 2021
OV-UCLA ERI
Institutional Review Board

a. No tengo una tarjeta de identificación (ID) ni una licencia de conducer
b. Temo que me hagan preguntas sobre mi estado migratorio.
c. Temo que tendré que darles demasiada información.
d. No tengo un médico de cabecera (doctor de primaria) ni una clínica
e. No tengo coche ni transporte
f. No se donde conseguirlo
g. No tengo acceso a Internet para obtener una cita
h. Temo que mis prospectos de inmigración se vean afectadas si me vacunan
i. Temo que me reporten a las autoridades de inmigración.
j. Otra:______________________________________

16. ¿Conoce a algún inmigrante indocumentado que tenga miedo de recibir la vacuna COVID porque cree que podría ser reportado a las autoridades?
    a. Si
        i. Si es así, ¿cuántos?_____________________
    b. no

If they have misconceptions, explain to them that undocumented immigrants can freely get COVID vaccines, that they do not ask about immigration status and do not report to immigration authorities.
