# Supplementary material for: COVID-19 Vaccine Uptake in Undocumented Latinx Patients Presenting to the Emergency Department
Source: JAMA Netw Open. 2024 Apr 26;7(4):e248578. doi: 10.1001/jamanetworkopen.2024.8578 (PMC11053375; doi:10.1001/jamanetworkopen.2024.8578)
Supplement: Supplement 2. — Data Sharing Statement [file jamanetwopen-e248578-s002.pdf]

Data Sharing Statement

Torres. COVID-19 Vaccine Uptake in Undocumented Latinx Patients Presenting to the Emergency Department. *JAMA Netw Open*. Published April 26, 2024. doi:10.1001/jamanetworkopen.2024.8578

Data
Data available: No
